# Using one health training for interprofessional team building: implications for research, policy, and practice in Nigeria

**DOI:** 10.3389/fpubh.2024.1375424

**Published:** 2024-07-30

**Authors:** Nathan Shehu, Pam Luka, Dennis Bente, Rebecca Weka, Caroline Weldon, Dung D. Pam, Simeon Cadmus, Filibus Dami, Slobodan Paessler, Scott Weaver, Matthew Dacso

**Affiliations:** ^1^West African Center for Emerging Infectious Diseases, Jos University Teaching Hospital, Jos, Nigeria; ^2^Department of Pathology, University of Texas Medical Branch, Galveston, TX, United States; ^3^National Veterinary Research Institute, Vom, Nigeria; ^4^Department of Microbiology and Immunology, University of Texas Medical Branch, Galveston, TX, United States; ^5^Department of Zoology, University of Jos, Jos, Nigeria; ^6^University of Ibadan, Oyo, Nigeria; ^7^Department of Veterinary, Public Health and Preventive and Centre for Control and Prevention of Zoonoses, University of Ibadan, Oyo, Nigeria; ^8^Department of Microbiology Nigerian Institute of Medical Research, Lagos, Nigeria; ^9^Department of Global Health and Emerging Diseases and Department of Internal Medicine. University of Texas Medical Branch, Galveston, TX, United States

**Keywords:** one-health approach, training, team building, Nigeria, inter-professional

## Abstract

In recent years, the concept of One Health (OH) has arisen as an approach that helps to catalyze the creation of transdisciplinary teams needed for surveillance and investigation of emerging disease dynamics. Besides a wealth of descriptions of what the OH approach encompasses, a dearth of information is available regarding the training of individuals in OH competencies. In 2019, the Nigerian Center for Disease Control developed an OH strategic plan to meet the country’s human, animal, and environmental health challenges. In response to the demand for clinicians, scientists, climatologists, conservationists, and environmentalists, who have expertise in environment, human, plant, and animal health to work collaboratively in addressing OH challenges in Nigeria. An interprofessional group of faculty from the University of Texas Medical Branch, the University of Jos, and the National Veterinary Research Institute convened to develop a novel OH course ‘entitled ‘One Health for Translational Team Science. The objective of the course was to explore the evolution of an emerging epidemic, capitalizing on various learning environments, including animal, environmental, human, and public health perspectives. The 6-week course comprised of three parts: 2-weeks virtual part of case-based group discussions focusing on animal and environmental aspects, 2 weeks of individual field experiences, and a final virtual part focusing on human health. Pedagogical tools used were: case-based group discussions, breakout group presentations, role-play activities, field project write-up, peer evaluation, group writing assignments, and weekly reflections with the goal of working in teams to develop and practice the fundamental leadership and management skills in addressing emerging public health challenges. Post-course evaluations showed that all participants felt more confident identifying and practicing the necessary attitudes and skills to participate effectively in the evaluation of an outbreak. Furthermore, the roles, responsibilities, and “One Health ways of thinking” for the various disciplines and professions involved in improving global health were articulated and identified.

## Background and rationale for the educational activity innovation

A growing global human population, destruction of ecosystems, and very close interactions between animals, humans and the environment threaten all those that share those ecosystems (1). The interactions of these factors have increased the risk of the emergence of infectious diseases and their transmission among humans and animals (2). Over the years, these interactions have led to the emergence of viruses such as COVID-19, Nipah, SARS, avian and swine flu, Ebola, Zika, and MERS, which are all of animal origin (zoonotic) and constitute a significant threat to global health (3, 4). Continuous environmental pollution and climate change also increase the emergence of infectious diseases (5–7).

Globally, approximately 70% of zoonotic and emerging infectious diseases originate in wild animals and thus can have a significant effect on the economy of nations (6, 8). The Ebola disease outbreak of 2014 cost approximately $12 billion to control, while the COVID-19 pandemic is reported to have cost more than $15.8 trillion globally (9, 10). These recent infectious disease outbreaks have exposed gaps in global health systems and structures designed earlier for disease prevention and control (11).

In recent years, the concept of One Health (OH) has arisen as an approach that helps to catalyze the creation of interdisciplinary teams needed for surveillance and investigation of emerging disease dynamics. One Health is defined as an “approach to designing and implementing programs, policies, legislation and research involving multisectoral groups that communicate and work together for better public health outcomes” (12). Proponents of OH have argued that elaborating interdisciplinary competencies and developing training programs are key elements for improving population health (13). Simply put, the complexity of current health challenges requires a highly diverse and well-trained health workforce that can work across disciplines and beyond conventional borders and boundaries (14, 15).

## Pedagogical framework(s)

**Figure 1:**
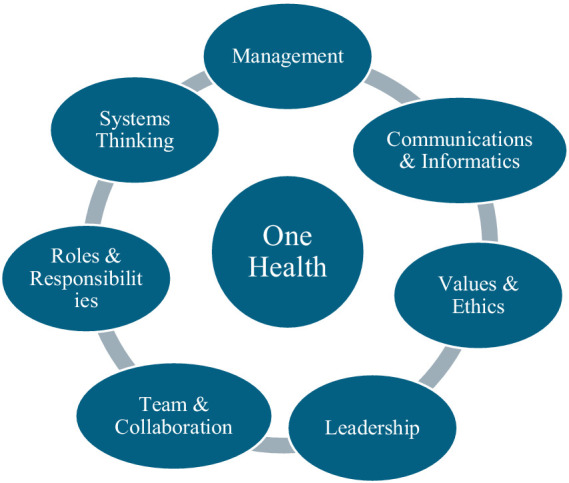
One health consensus competencies. Adapted from (14).

Despite a clear need for improved interprofessional collaboration to meet complex modern public health challenges, most health professions continue to train in silos (16, 17). Adopting a OH approach to training requires sharing knowledge to create truly transdisciplinary curricula. In 2016, a high-level panel was convened to synthesize the results of several high-level One Health curriculum design meetings and develop consensus One Health competencies (14). These are summarized in Figure 1.

Adopting an OH approach offers many benefits. These include improved diagnosis and prevention of infectious diseases transmitted between animals and humans through the environment; improvement in the management of domesticated animals and the psychosocial status of patients; and early detection of health hazards (18, 19).

Therefore, OH interventions often cut across multiple disciplines: medical, veterinary, environmental, and other relevant fields and sectors (1). These interdisciplinary collaborations are needed to understand the dynamics of human-animal population migration, habitat destruction, and interaction at the human-animal interface. For example, human population growth has resulted in expansion of activities into areas initially considered not conducive for human habitation, thereby causing interactions between domestic and mostly wild animals which predisposed to risk of infectious disease transmission between and among species (6, 8).

### Competencies/standards underlying the educational activity

In 2016, the continued emergence of infectious diseases led the Food and Agriculture Organization (FAO), World Organization for Animal Health (WOAH, founded as OIE), World Health Organization (WHO), and other United Nations agencies to adopt a strategy focused on reducing risks of infectious diseases at the animal-human-ecosystems interface by employing an OH approach for disease control (20). In 2020, the National Institute of Allergy and Infectious Diseases (NIAID) of the NIH set up 10 Centers for Research in Emerging Infectious Diseases (CREID), including our West African Center for Emerging Infectious Diseases (WAC-EID) for the detection of early warning signs at the human-animal interface. The WAC-EID uses the OH approach in disease outbreak investigation, surveillance, and diagnosis.

In 2019, the Nigerian Center for Disease Control (NCDC) developed an OH strategic plan to meet the country’s human, animal, and environmental health challenges (21). This is in line with the mandate of African Center for Disease Control, to strengthen the capacities, capabilities and partnerships of African countries Africa (22). The approach aims to address health challenges by leveraging synergies across ministries, health institutions, and related disciplines. The strategic plan aims to improve inter-sectoral communication, generate evidence-based solutions, train a new generation of systems-thinkers, enhance surveillance and outbreak investigation, decrease lag time in response, and improve health and economic savings. However, barriers to the operationalization of this approach include perception and cultural differences, competition over budgets, communication challenges, and the need for improved technology for interconnection (11, 23).

The objectives, course activities, and methods of assessment of the course are summarized in Table 1. This training complements other existing OH training programs, it is in response to the demand for clinicians, scientists, and public health practitioners to work collaboratively to address OH challenges in Nigeria. There were several parallel profession-tailored trainings related to public health in Nigeria. An inter-professional group of faculty from the University of Texas Medical Branch (UTMB), the Jos University Teaching Hospital, the University of Jos, and the National Veterinary Research Institute (NVRI) convened to develop a novel OH course, “One Health for Translational Team Science.,” and the rationale and local relevance of the objectives are further explored in the Discussion Section. There were senior faculty among the trainees from biological science, environmental science, medicine, and veterinary science. The trajectory is this would eventually be incorporated into the curriculum of the University of Jos as a pilot in Nigerian Universities.

**Table 1:** Training objectives, activities, and methods of assessment.

Objective	Course activity	Method of assessment
Describe the influence that social, economic, environmental, and cultural determinants of population health have on the emergence of novel pathogens	Case-based group discussions Individual field experiences	Writing assignments Field project write up
Identify and practice the necessary attitudes and skills to participate effectively in the evaluation of an outbreak	Role-play activity Field project	Peer evaluation and feedback
Define the roles, responsibilities, and “ways of thinking” for the various disciplines and professions involved in improving global health	Field project Case-based group discussions	Writing assignments Weekly reflections
Work in teams to develop and practice the fundamental leadership and management skills involved in addressing emerging public health challenges	Case-based group discussions Breakout group presentations Role-play activity	Field project write up
Explore the evolution of an emerging epidemic capitalizing on a variety of learning environments, including animal, environmental, human, and public health perspectives	Individual field experiences Role-play activity Case-based group discussions	Field project write up

As part of the course, a questionnaire was distributed to collect data on participants’ knowledge regarding the existence and implementation of OH actions and initiatives in Nigeria. A pre-post evaluation was also administered to evaluate participants’ understanding of OH concepts and approaches before and after attending the OH course. Next, we describe the structure of the course and the results of these evaluations.

### Learning environment (setting, students, faculty)

The “One Health for Translational Team Science” course was a strategic health manpower capacity development training program was organized for both junior and senior faculty of Jos University Teaching Hospital (JUTH), National Veterinary Research Institute (NVRI) Vom, the University of Ibadan, and the University of Jos in Nigeria.

#### Pedagogical format

**Figure 2:**
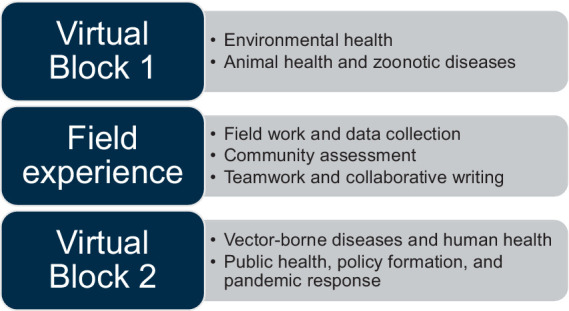
One health course training structure.

The training structure is summarized in Figure 2. The course was divided into 3 phases: first, a 2-week block of virtual sessions, followed by a 2-week block for group field activities and writing assignments, and finally a second 2-week block of virtual sessions. Each week, the virtual sessions included two 2-h, synchronous case-based interactive discussions, independent field activities, and asynchronous group discussion of assignments. Problem-based learning approaches and small-group breakouts were used in the synchronous teaching sessions to optimize group participation. Rotating small group “leaders” presented on behalf of their groups during the virtual sessions, giving all members an opportunity to practice public speaking and communication skills.

## Methods

**Figure 3:**
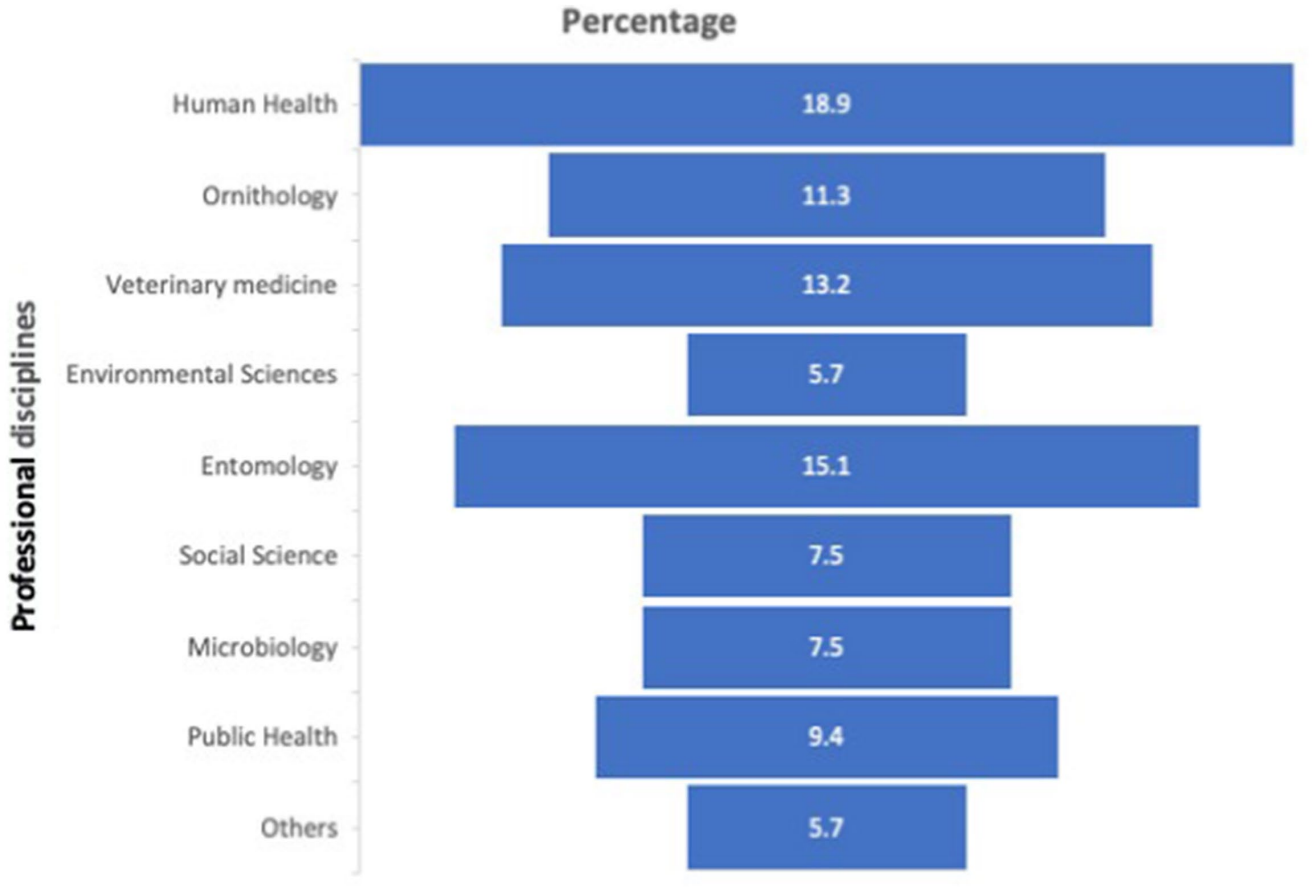
Distribution of professional disciplines of the trainees. Others: Mammologists, and those not indicated.

The OH training was conducted between September and October 2021. A multi-disciplinary cohort was purposively selected from 5 tertiary institutions in Nigeria, the University of Jos, Jos University Teaching Hospital, A. P Leventis Ornithological Research Institute, the University of Ibadan, and the National Veterinary Research Institute Vom. Two faculty from the UTMB OH program, a veterinarian, and a medical doctor, served as the principal course directors. Three Nigerian faculty members, a medical doctor, a veterinarian, and an entomologist, served on the course planning committee. A hybrid virtual/field-based approach accommodated constraints imposed by the COVID-19 pandemic as well as geographic and time zone differences. Given the need for participants to join from diverse institutions, the Top Hat learning management interface combined with Zoom platform were used for the virtual training sessions. To optimize interprofessional interactions, participants were intentionally assigned to 10-member multidisciplinary (e.g., clinicians, veterinarians, entomologists) groups for the duration of the course (Figure 3).

### Evaluation and pre-post testing

The survey tool used for the pre-post testing was adapted from an existing OH survey tool (24). Questions were incorporated in the course’s pre-test to explore existing collaboration among the animal health, human, public health, and environmental health sectors, and to collect information on the existence and implementation of OH actions and initiatives in Nigeria. Evaluation of OH knowledge of participants before and after the course involved a 4-point Likert scale that rated everyone’s level of confidence ranging from 1 (not confident), 2 (Somewhat confident), 3 (Confident) and 4 (Very confident) in the course objectives. The mean scores and standard deviation were compared before (pre) and after the training to determine the difference in confidence of knowledge gain. The survey was implemented in RedCap, a web-based application developed by Vanderbilt University to capture data for clinical research and create databases and projects (25). It is Health Insurance Portability and Accountability Act (HIPAA)–compliant, highly secure and intuitive to use. The participants took 10–15 min to complete the survey.

### Data collation and analysis

Data were entered into an Excel spreadsheet (Microsoft Inc.) and exported into IBM SPSS Statistics for Windows, Version 23 (IBM Corp., Armonk, N.Y., United States) for analysis. The percentage distribution of the test results and questionnaire data were generated, and frequency tables were done to quantify the outcome of responses. Student’s T-test was used to determine differences between pre and post-test scores. Results were presented as tables and figures. All *p*-values were two-tailed and statistical significance was designated <0.05 at 95% CI.

## Results to date/assessment

### Data already gathered

*Participants and Pre-Test Results.* There were 53 participants: 75.5% male and 25.5% female, 10 (19%) medical doctors, 8 (15%) entomologists, and 7 (13%) veterinary doctors; details of the distribution of professional backgrounds are presented in Figure 3. Twenty-seven (51%) participants had the highest earned degree of master’s. Six (11%) of the participants were senior faculty and leaders, this includes readers, professors, a Director, and a Dean.

*Pre and Post-Test Results.* On the pre-course assessment, 16 (25%) of the participants had never heard of OH; 30 (57%) and 7 (13.2%) rated awareness/perception of critical OH stakeholders in Nigeria as low and moderate, respectively. About 37.7% of the males and 17% of females reported One Health initiatives were implemented in the country. Twenty-two (42%) of participants were not aware that OH was being implemented in Nigeria. Nine (17%) did not know and 23 (43%) were unsure if there were formal connections between veterinary/animal health and public health administration in Nigeria. Only 19 (36%) were aware of the existence of boards/committees/associations actively dealing with OH issues/initiatives in Nigeria.

The combined participants groups’ field activities and knowledge and application of OH among OH training participants are presented in Table 2. Overall, there was a statistically significant improvement in knowledge of and confidence in the application of OH among participants after the training. Participants’ answers significantly improved on all questions. The participants were divided into 5 subgroups; each group carried out field activities at different locations. The summary of the field experience component of the training is presented in Table 3. The fieldwork provided an opportunity for multi-specialty participants to work together in teams, develop field activity objectives, implement the fieldwork and provided recommendations.

**Table 2:** Pre-post testing results: knowledge and application of one health among Nigerian participants.

S/N	Questions	Pre-test mean score (SD)	Post-test mean score (SD)	*t*-Score	*p*-value
1	Describe the influence that social, economic, environmental, and cultural determinants of population health have on the emergence of novel pathogens.	2.98 (0.85)	3.42 (0.72)	−3.195	0.002
2	Identify and practice the necessary attitudes, and skills to participate effectively in the evaluation of an outbreak.	2.89 (0.89)	3.32 (0.64)	−3.017	0.004
3	Define the roles, responsibilities, and ‘ways of thinking’ for the various disciplines and professions involved in improving global health.	2.89 (0.95)	3.28 (0.69)	−2.586	0.013
4	Work in teams to develop and practice the fundamental leadership and management skills involved in addressing emerging public health challenges.	3.02 (0.84)	3.58 (0.60)	−4.006	< 0.001
5	Explore the evolution of an emerging epidemic from a variety of learning environments, including animal, environmental, human, and public health perspectives.	2.85 (0.91)	3.43 (0.67)	−4.072	< 0.001

**Table 3:** The fieldwork activity of a representative participant.

Groups	Objectives	Location of field activity	Field work result	Recommendation
1 (Nest)	1. To conduct an outbreak investigation post African swine fever (ASF) outbreak. 2. To determine the possible factors related to the outbreak 3. To determine if the food processing facilities of the farm were sanitary.	1. Swine farm, Jos Plateau State 2. Abattoir, Jos Plateau state	1. Economic losses due to the outbreak. 2. Mixed farming of livestock 3. Some unhealthy food processing practices were observed such as use of plastics and petrol for meat processing.	1. Training, advocacy, and routine inspection of meat processing. 2. Discourage mixed farming with swine to prevent pathogen amplification.
2 (Dove)	1. To undertake post African Swine fever (ASF) outbreak investigation. 2. To determine one-health gaps in the farm	Swine farm, Jos Plateau State	1. Significant economic loss due to the ASF that affected the farm 2. Poor refuse disposal 3. Rodent trapping, sample yielded *Escherichia coli* (confirmed by PCR) 3. Mixed farming of animals in close proximity (e.g., swine, birds): potential for pathogen amplification and transmission to humans 4. Nearby unhygienic pond used by animals. Water sample analysis yielded *Escherichia coli* and *Aeromonas* spp.	1. Intensify efforts to prevent, detect, and respond to ASF. 2. Advocacy, policy formulation, implementation, and robust monitoring of farms to ensure compliance with animal and environmental sanitation.
3	1. Perform a systematic review to understand the socio-cultural, religious, economic, and political factors that create an enabling environment for rabies to thrive and continue to be a public health challenge in Nigeria. 2. To find and highlight the gaps in knowledge and practice of the local population in dog maintenance and care, and in the practice of diverse experts who are stakeholders in the prevention and management of the spread of rabies.	Not applicable	1. We found that poverty and high crime rates in communities are associated with a high incidence of rabies. 2. Sociocultural factors such as comfort with free roaming of dogs was also found to be associated with a high incidence of rabies. 3. Finally, economic and political factors such as political will and release of resources to support community-based programs for the control of rabies was also identified as a significant factor affecting the incidence of rabies.	1. The culture of allowing free roaming dogs should be discouraged in society. 2. The government should show commitment in supporting community-based programs to control rabies in endemic communities. 3. The government should put measures in place to reduce poverty and ensure security of lives and property, as this could be helpful in reducing the incidence of rabies. 4. Education of affected communities should be performed to help them take appropriate measures to prevent and treat this disease.
4	1. To determine the origin of the cases of rabies. 2. To trace all cases of dog bites from rabid dogs in the identified community.	Toro LGA, Bauchi State	1. The affected dogs were from a community in Toro LGA of Bauchi state. 2. Two individuals were bitten by rabid dogs in the community. They had not received post-exposure prophylaxis as at the time of the study.	1. We recommended that all affected persons should receive post-exposure prophylaxis, which was done. 2. Vaccination of all dogs in the affected community was recommended, for which the Director Veterinary Services from the State Ministry of Agriculture immediately supplied dog anti rabies vaccines for vaccination of all dogs in that community.
5	1. Observe abattoir environment for potential disease risk to animals and human populations 2. Identify One Health risk gaps	Abattoir, Jos Plateau State	1. No prior screening / testing of animals by specialists before slaughtering 2. The personnel and meat processors not well kitted (coats, gloves, boots, masks etc.) 3. No safe and portable water supplies 4. Slaughtered animals were burnt with old, discarded automobile tires, yielding wild flames and thick black smoke. This is a practice we considered hazardous for abattoir staff, considering the toxic emissions, hydrocarbons, benzene fumes that may be carcinogenic and harmful to the respiratory tract over time. In addition, scald burns are a potential hazard.	1. Regular screening and testing of meat and staff to prevent disease outbreaks 2. There should be aggressive sustainable public health campaigns by trained personnel using validated and standardized modules and materials for the abattoir staff

## Discussion on the practical implications

We present an assessment of OH training carried out in North-central Nigeria. Before the training, we found low knowledge and awareness of the implementation of OH in Nigeria. Despite the well-articulated Nigeria policy on OH (21), gaps remained in knowledge and implementation. The OH policy document is a 5-year strategic plan that ended in the year 2023. Since the rounding up of this policy document, it is necessary to evaluate and further strategize in formulating the next 5-year strategic plan. Thus, each course objectives were based on Nigeria’s OH policy and on local, national, and global OH priorities.

### Objectives, and lessons learned

The post-training assessment of the objective “Describe the influence that social, economic, environmental, and cultural determinants of population health have on the emergence of novel pathogens” was significantly higher than the pre-training assessment. This result is important not only because emerging diseases have a significant influence on human societies globally, affecting present and future generations, but also because changes in human living patterns, along with environmental and climate changes, pose unprecedented challenges to the global health of people, animals, and ecosystems (26–28). The result is also locally relevant to Nigeria, which is experiencing growing advances of humans and livestock into wildlife habitats, rising movement of wildlife from ecologically degraded areas into urban and peri-urban regions, massive gathering of people moving into densely populated cities, and rapid global movement of humans, animals, and their products. Some of Nigeria’s urban areas are also experiencing abandonment and consequent “re-greening,” which can also be a risk to public health. Abandonment increases zoonotic vector-borne disease risk by promoting the hyperabundance of commensal pests and pathogen vectors, while “greening” can increase transmission risk by promoting the spread of pathogen vectors between fragmented areas and by increasing novel human-wildlife interfaces.

Trainees were more confident after the training in Objective 2: identifying and practicing the necessary attitudes and skills to participate effectively in the evaluation of an outbreak. This indicates that the training was an effective method for the OH course and will improve the knowledge/skills of a multidisciplinary team (Objective 4). However, this finding may be mitigated by the findings of Mollon et al. (29) which found that online education was not a significant predictor of practice, attitudes, or knowledge/skills regarding evidence-based practice. It is important to note, however, that the Mollon study was conducted before the COVID pandemic forced greater reliance on online training, which may have increased both the quality of the training and the participants’ ability to apply it. In addition, our training was only partially online. It also included significant practical, in-person learning experiences.

The mean score post-test for Objective 3: “Define the roles, responsibilities, and ‘ways of thinking’ for the various disciplines and professions involved in improving global health” was higher than the mean score for the pre-test response and the difference was significant. This objective supports an institutional framework that has been proposed for emerging zoonotic diseases that require a multidisciplinary, “One Health” approach for their management (30). This approach recognizes the interdependence of human health, animals, and ecosystems, necessitating superior support for novel methodologies across disciplines combining quantitative and qualitative methods that also directly address the politics of policy processes (28). Previous authors have suggested the need for a concept that precisely addresses the development of a cohesive practice among different professionals from the same or different organizations and factors influencing it (31). The same authors also suggest that interprofessional education requires collaborative practice settings such as ours. Zwarenstein et al. (32) also reported that collaborative practice interventions indeed improve patient outcomes.

Our participants were more confident after the training in exploring the evolution of an emerging epidemic from a variety of learning environments, including animal, environmental, human, and public health perspectives (Objective 5). This object is rooted in reports such as Cutler et al. (33) that factors prompting emerging and reemerging zoonoses in human or animal infection include climate change influencing arthropods, translocation of infected animals or persons, tourism, variations in land use, adaptation of pathogens to new host species, acquisition of unique virulence traits, changes in livestock management systems, companion animals, foods (bush meat), and exotic pets.

The uniqueness of the training included the multi-professional participants, problem-based learning, and field group activities. Employing diverse, active learning methods may have contributed to the enthusiasm of participants and significant objective improvement in learning outcomes. Previous literature suggests that there is enhanced learning and real-life application of knowledge when fieldwork activities are incorporated into teaching (34). The training is geared toward equipping trainees with skills that they will then use in their current jobs.

This course also incorporated several educational innovations. Since the course took place during the COVID-19 pandemic, the organizers had to create virtual and field-based learning environments that optimized the safety of participants and maintained adequate social distancing. Discussion groups were intentionally selected to ensure diverse representation of different professional backgrounds and to create camaraderie among participants. Active learning methodologies were incorporated throughout the course to maximize the amount of interprofessional interaction. During the planning process, the curriculum was co-designed by an interprofessional group of Nigerian and US veterinary and medical doctors, ecologists who were educators, and researchers to ensure that course content was culturally and contextually appropriate.

This training provided the opportunity to learn team dynamics in OH. The group activities included outbreak investigation, meat processing, OH environmental assessment, and zoonoses. These are undoubtedly critical areas of OH and the experience gained would significantly help implement OH policies and programs. The obvious challenge of OH implementation is the vertical nature of the Nigerian Ministries of Health, Environment, and Agriculture.

### Acknowledgment of any conceptual, methodological, environmental, or material constraints

The generalizability of our study is limited because the number of participants in the course was much fewer than the number of relevant personnel in Nigeria. This course nonetheless represents a springboard toward the scaling up of training of OH in Nigeria. Additionally, the team’s composition was not perfectly matched with equal numbers of each category of professionals, but most are represented. The long-term outcome measure of this training of institutionalizing this training into institutions in Nigeria was not determined because it requires long-term of planning, advocacy, approvals, and curriculum harmonization. However, after the training, an inter-departmental implementation committee was formed. We have not addressed Other OH competency frameworks like Tripartite Competencies for One Health field epidemiology (COHFE) Network for Ecohealth and One Health (NEOH), Africa CDC, and Core Competencies for One Health Education (16, 22, 35). Frankson et al. OH competency framework was used, which has some agreement with the framework of the Network for Ecohealth and One Health (NEOH).

Overall, the “One Health for Translational Team Science” course demonstrated excellent learning outcomes. Employing a virtual/hybrid format carries the potential to widen and deepen the impact of the training. The results of this study demonstrate the importance of bringing together the multiple structures and professional bodies that are working on OH in Nigeria to optimize synergies and minimize duplication of effort. This course represents a significant opportunity to strengthen the OH workforce and move further toward the implementation of OH policies and programs in Nigeria. As international efforts increase to address emerging threats to global public health, similar training may be adopted in other countries.

## Data availability statement

The original contributions presented in the study are included in the article/Supplementary material, further inquiries can be directed to the corresponding author.

## Ethics statement

The studies involving humans were approved by National Health Research Ethics Committee of Nigeria (NHREC/01/01/2007–11/12/2020). The studies were conducted in accordance with the local legislation and institutional requirements. The participants provided their written informed consent to participate in this study.

## Author contributions

NS: Conceptualization, Data curation, Funding acquisition, Methodology, Project administration, Resources, Supervision, Validation, Writing – original draft, Writing – review & editing. PL: Conceptualization, Funding acquisition, Methodology, Project administration, Resources, Supervision, Writing – original draft, Writing – review & editing. DB: Conceptualization, Methodology, Project administration, Resources, Supervision, Writing – original draft, Writing – review & editing. RW: Data curation, Methodology, Writing – original draft, Writing – review & editing. CW: Conceptualization, Formal analysis, Methodology, Project administration, Resources, Supervision, Writing – original draft, Writing – review & editing. DP: Conceptualization, Funding acquisition, Project administration, Supervision, Writing – original draft, Writing – review & editing. SC: Writing – original draft, Writing – review & editing. FD: Conceptualization, Project administration, Supervision, Writing – original draft, Writing – review & editing. SP: Writing – review & editing, Conceptualization, Funding acquisition, Project administration, Resources, Supervision, Validation, Writing – original draft. SW: Conceptualization, Project administration, Resources, Supervision, Writing – original draft, Writing – review & editing, Funding acquisition, Validation. MD: Conceptualization, Data curation, Formal analysis, Methodology, Project administration, Resources, Supervision, Writing – original draft, Writing – review & editing.
