# Supplementary material for: Using one health training for interprofessional team building: implications for research, policy, and practice in Nigeria
Source: Front Public Health. 2024 Jul 30;12:1375424. doi: 10.3389/fpubh.2024.1375424 (PMC11323119; doi:10.3389/fpubh.2024.1375424)
Supplement: Supplementary file 1 [file Table_1.DOCX]

SUPPLEMENTARY TABLES:

**Supplementary Table I. Characteristics of participants according to gender and profession**

| Gender |  | Male | % | Female | % | Total | % |
| --- | --- | --- | --- | --- | --- | --- | --- |
| Disciplinary background? | Human Health | 9 | 17 | 1 | 1.9 | 10 | 18.9 |
|  | Ornithology | 5 | 9.4 | 1 | 1.9 | 6 | 11.3 |
|  | veterinary medicine | 5 | 9.4 | 2 | 3.8 | 7 | 13.2 |
|  | Environmental Sciences | 3 | 5.7 | 0 | 0 | 3 | 5.7 |
|  | Entomology | 6 | 11.3 | 2 | 3.8 | 8 | 15.1 |
|  | Social Science | 2 | 3.8 | 2 | 3.8 | 4 | 7.5 |
|  | Microbiology | 3 | 5.7 | 1 | 1.9 | 4 | 7.5 |
|  | Public Health | 3 | 5.7 | 2 | 3.8 | 5 | 9.4 |
|  | other | 4 | 7.5 | 2 | 3.8 | 6 | 11.3 |
|  | Total | 40 | 75.5 | 13 | 24.5 | 53 | 100 |
| Highest university degree title | PhD | 16 | 13.2 | 2 | 3.8 | 18 | 34 |
|  | MSc | 17 | 32.1 | 8 | 15.1 | 25 | 47.2 |
|  | Undergraduate | 5 | 9.4 | 2 | 3.8 | 7 | 13.2 |
|  | Diplomas | 2 | 3.8 | 1 | 1.9 | 3 | 5.7 |
|  | Total | 40 | 75.5 | 13 | 24.5 | 53 | 100 |
| Institution employed | Human Health | 8 | 15.1 | 1 | 1.9 | 9 | 17 |
|  | Animal Science | 5 | 9.4 | 3 | 5.7 | 8 | 15.1 |
|  | WAC-EID | 1 | 1.9 | 0 | 0 | 1 | 1.9 |
|  | Environmental Sciences | 7 | 13.2 | 1 | 1.9 | 8 | 15.1 |
|  | Research Institute | 11 | 20.8 | 0 | 0 | 11 | 20.8 |
|  | Public Health | 5 | 9.4 | 4 | 7.5 | 9 | 17 |
|  | University | 3 | 5.7 | 1 | 1.9 | 4 | 7.5 |
|  | Other | 0 | 0 | 2 | 3.8 | 2 | 3.8 |
|  | Non-governmental organization | 0 | 0 | 1 | 1.9 | 1 | 1.9 |
|  | Total | 40 | 75.5 | 13 | 24.5 | 53 | 100 |
| Present position | Not indicated | 0 | 0 | 1 | 1.9 | 1 | 1.9 |
|  | Reader&co | 4 | 7.5 | 0 | 0 | 4 | 7.5 |
|  | Research assistant | 7 | 13.2 | 4 | 7.5 | 11 | 20.8 |
|  | Consultant &co | 3 | 5.7 | 0 | 0 | 3 | 5.7 |
|  | Project Manager | 0 | 0 | 1 | 1.9 | 1 | 1.9 |
|  | Principal Veterinary research Officer | 3 | 5.7 | 0 | 0 | 3 | 5.7 |
|  | Chief Vet. Research Officer | 3 | 5.7 | 1 | 1.9 | 4 | 7.5 |
|  | Professor | 2 | 3.8 | 0 | 0 | 2 | 3.8 |
|  | Medical Superintendent | 5 | 9.4 | 1 | 1.9 | 6 | 11.3 |
|  | Senior Research Officer | 1 | 1.9 | 0 | 0 | 1 | 1.9 |
|  | Director | 1 | 1.9 | 1 | 1.9 | 2 | 3.8 |
|  | Program coordinator | 0 | 0 | 1 | 1.9 | 1 | 1.9 |
|  | Lecturer II/ Research Associate | 7 | 13.2 | 1 | 1.9 | 8 | 15.1 |
|  | Entomology technician | 4 | 7.5 | 2 | 3.8 | 6 | 11.3 |
|  | Total | 40 | 75.5 | 13 | 24.5 | 53 | 100 |
| One Health initiatives being implemented in your country | Yes | 20 | 37.7 | 9 | 17 | 29 | 54.7 |
|  | No | 20 | 37.7 | 4 | 7.5 | 24 | 45.3 |
|  | Total | 40 | 75.5 | 13 | 24.5 | 53 | 100 |

**Supplementary Table II. Personal opinion, knowledge and perception of participants on One Health initiatives**

**being implemented in the country according to disciplinary background**

|  |  | H H | Ornit | vet | Env Scie | Ento | SocSci | Micro | PH | other | Total |
| --- | --- | --- | --- | --- | --- | --- | --- | --- | --- | --- | --- |
| Are there any One Health initiatives being implemented in your country? | Yes | 7(`13.2) | 2(3.8) | 6(11.3) | 1(1.9) | 3(5.7) | 2(3.8) | 3(5.7) | 1(1.9) | 4(7.5) | 29(54.7) |
|  | No | 3(5.7) | 4(7.5) | 1(1.9) | 2(3.8) | 5(9.4) | 2(3.8) | 1(1.9) | 4(7.5) | 2(3.8) | 24(45.3) |
| Total |  | 10(18.9) | 6(11.3) | 7(`13.2) | 3(5.7) | 8(15.1) | 4(7.5) | 4(7.5) | 5(9.4) | 6(11.3) | 53(100) |
| Disease Surveillance and monitoring) | yes | 5(9.4) | 2(3.8) | 6(11.3) | 1(1.9) | 2(3.8) | 1(1.9) | 1(1.9) | 1(1.9) | 3(5.7) | 22(41.5) |
|  | No | 5(9.4) | 4(7.5) | 1(1.9) | 2(3.8) | 6(11.3) | 3(5.7) | 3(5.7) | 4(7.5) | 3(5.7) | 31(58.5) |
| Total |  | 10(18.9) | 6(11.3) | 7(`13.2) | 3(5.7) | 8(15.1) | 4(7.5) | 4(7.5) | 5(9.4) | 6(11.3) | 53(100) |
| Disease prevention and control | Yes | 5(9.4) | 0(0) | 5(9.4) | 1(1.9) | 2(3.8) | 2(3.8) | 1(1.9) | 1(1.9) | 2(3.8) | 19(35.8) |
|  | No | 5(9.4) | 6(11.3) | 2(3.8) | 2(3.8) | 6(11.3) | 2(3.8) | 3(5.7) | 4(7.5) | 4(7.5) | 34(64.2) |
| Total |  | 10(18.9) | 6(11.3) | 7(`13.2) | 3(5.7) | 8(15.1) | 4(7.5) | 4(7.5) | 5(9.4) | 6(11.3) | 53(100) |
| Awareness of participants of programs) | Yes | 3(5.7) | 0(0) | 1(1.9) | 0(0) | 1(1.9) | 1(1.9) | 0(0) | 0(0) | 0(0) | 6(11.3) |
|  | No | 7(`13.2) | 6(11.3) | 6(11.3) | 3(5.7) | 7(`13.2) | 3(5.7) | 4(7.5) | 5(9.4) | 6(11.3) | 47(88.7) |
| Total |  | 10(18.9) | 6(11.3) | 7(`13.2) | 3(5.7) | 8(15.1) | 4(7.5) | 4(7.5) | 5(9.4) | 6(11.3) | 53(100) |
| Higher Education programs) | Yes | 3(5.7) | 0(0) | 1(1.9) | 0(0) | 1(1.9) | 0(0) | 0(0) | 0(0) | 0(0) | 5(9.4) |
|  | No | 7(`13.2) | 6(11.3) | 6(11.3) | 3(5.7) | 7(`13.2) | 4(7.5) | 4(7.5) | 5(9.4) | 6(11.3) | 48(90.6) |
| Total |  | 10(18.9) | 6(11.3) | 7(`13.2) | 3(5.7) | 8(15.1) | 4(7.5) | 4(7.5) | 5(9.4) | 6(11.3) | 53(100) |
| Research | Yes | 3(5.7) | 2(3.8) | 4(7.7) | 1(1.9) | 1(1.9) | 1(1.9) | 3(5.7) | 0(0) | 2(3.8) | 17(32.7) |
|  | No | 6(11.3) | 4(7.7) | 3(5.7) | 2(3.8) | 7(13.5) | 3(5.7) | 1(1.9) | 5(9.6) | 4(7.7) | 35(67.3) |
| Total |  | 9(17.3) | 6(11.3) | 7(`13.2) | 3(5.7) | 8(15.1) | 4(7.7) | 4(7.7) | 5(9.6) | 6(11.3) | 52(100) |
| FORMAL connections between veterinary/animal health and public health administration in your country (governmental institutions or services)? | Yes | 6(11.3) | 3(5.7) | 6(11.3) | 2(3.8) | 2(3.8) | 1(1.9) | 1(1.9) | 1(1.9) | 3(5.7) | 25(42.7) |
|  | No | 0(0) | 2(3.8) | 1(1.9) | 0(0) | 4(7.5) | 0(0) | 0(0) | 0(0) | 0(0) | 7(13.2) |
|  | Unsure | 4(7.5) | 1(1.9) | 0(0) | 1(1.9) | 2(3.8) | 3(5.7) | 3(5.7) | 4(7.5) | 3(5.7) | 21(39.6) |
| Total |  | 10(18.9) | 6(11.3) | 7(`13.2) | 3(5.7) | 8(15.1) | 4(7.5) | 4(7.5) | 5(9.4) | 6(11.3) | 53(100) |
| At National level? | Yes | 6(11.3) | 3(5.7) | 5(9.4) | 1(1.9) | 2(3.8) | 1(1.9) | 1(1.9) | 1(1.9) | 3(5.7) | 23(48.4) |
|  | No | 4(7.5) | 3(5.7) | 2(3.8) | 2(3.8) | 6(11.3) | 3(5.7) | 3(5.7) | 4(7.5) | 3(5.7) | 30(56.6) |
| Total |  | 10(18.9) | 6(11.3) | 7(`13.2) | 3(5.7) | 8(15.1) | 4(7.5) | 4(7.5) | 5(9.4) | 6(11.3) | 53(100) |
| At Sub-national i.e. regional, provincial level | Yes | 3(5.7) | 0(0) | 2(3.8) | 1(1.9) | 1(1.9) | 1(1.9) | 0(0) | 0(0) | 2(3.8) | 10(18.9) |
|  | No | 7(`13.2) | 6(11.3) | 5(9.4) | 2(3.8) | 7(`13.2) | 3(5.7) | 4(7.5) | 5(9.4) | 4(7.5) | 43(81.1) |
| Total |  | 10(18.9) | 6(11.3) | 7(`13.2) | 3(5.7) | 8(15.1) | 4(7.5) | 4(7.5) | 5(9.4) | 6(11.3) | 53(100) |
| At Local level | Yes | 2(3.8) | 0(0) | 0(0) | 0(0) | 0(0) | 0(0) | 0(0) | 0(0) | 1(1.9) | 3(5.7) |
|  | No | 8(15.1) | 6(11.3) | 7(`13.2) | 3(5.7) | 8(15.1) | 4(7.5) | 4(7.5) | 5(9.4) | 5(9.4) | 50(94.3) |
| Total |  | 10(18.9) | 6(11.3) | 7(`13.2) | 3(5.7) | 8(15.1) | 4(7.5) | 4(7.5) | 5(9.4) | 6(11.3) | 53(100) |
| Boards/committees/associations actively dealing with One Health issues/initiatives in the country? | Yes | 3(5.7) | 0(0) | 4(7.5) | 1(1.9) | 2(3.8) | 1(1.9) | 3(5.7) | 0(0) | 3(5.7) | 17(32.1) |
|  | No | 1(1.9) | 0(0) | 0(0) | 0(0) | 1(1.9) | 0(0) | 0(0) | 2(3.8) | 0(0) | 4(7.5) |
|  | Unsure | 6(11.3) | 6(11.3) | 3(5.7) | 2(3.8) | 5(9.4) | 3(5.7) | 1(1.9) | 3(5.7) | 3(5.7) | 32(60.4) |
| Total |  | 10(18.9) | 6(11.3) | 7(`13.2) | 3(5.7) | 8(15.1) | 4(7.5) | 4(7.5) | 5(9.4) | 6(11.3) | 53(100) |
| Currently involved in One Health initiatives (e.g. surveillance, prevention and control of: vector-borne zoonoses; environmental containments in food; rabies; etc.) | Yes | 8(15.1) | 4(7.5) | 6(11.3) | 0(0) | 8(15.1) | 1(1.9) | 3(5.7) | 2(3.8) | 6(11.3) | 38(71.7) |
|  | No | 2(3.8) | 2(3.8) | 1(1.9) | 3(5.7) | 0(0) | 3(5.7) | 1(1.9) | 3(5.7) | 0(0) | 15(28.3) |
| Total |  | 10(18.9) | 6(11.3) | 7(`13.2) | 3(5.7) | 8(15.1) | 4(7.5) | 4(7.5) | 5(9.4) | 6(11.3) | 53(100) |
| One Health initiatives being implemented at your institution | Yes | 4(7.5) | 3(5.7) | 5(9.4) | 0(0) | 2(3.8) | 1(1.9) | 4(7.5) | 0(0) | 3(5.7) | 22(41.5) |
|  | No | 4(7.5) | 2(3.8) | 0(0) | 1(1.9) | 4(7.5) | 1(1.9) | 0(0) | 4(7.5) | 3(5.7) | 19(35.8) |
|  | Unsure | 2(3.8) | 1(1.9) | 2(3.8) | 2(3.8) | 2(3.8) | 2(3.8) | 0(0) | 1(1.9) | 0(0) | 12(22.6) |
| Total |  | 10(18.9) | 6(11.3) | 7(`13.2) | 3(5.7) | 8(15.1) | 4(7.5) | 4(7.5) | 5(9.4) | 6(11.3) | 53(100) |

**Suplementary Tables III. Participants pre training assesment**

|  |  | HH | Ornit | vet med | Envi Sci | Ento | Soc Scie | Micro | P H | other |  |
| --- | --- | --- | --- | --- | --- | --- | --- | --- | --- | --- | --- |
| Soc, eco, enviro, & cultural determinants of pop health have on the emergence of novel pathogens. | very confident | 3(5.7) | 3(5.7) | 3(5.7) | 0(0) | 2(2.8) | 0(0) | 2(2.8) | 2(2.8) | 0(0) | 15(28.3) |
|  | confident | 5(9.4) | 3(5.7) | 4(7.5) | 2(2.8) | 6(11.3) | 4(7.5) | 2(2.8) | 3(5.7) | 6(11.3) | 35(66) |
|  | Not confident | 2(2.8) | 0(0) | 0(0) | 1(1.9) | 0(0) | 0(0) | 0(0) | 0(0) | 0(0) | 3(5.7) |
| Total |  | 10(18.9) | 6(11.3) | 7(13.2) | 3(5.7) | 8(15.1) | 4(7.5) | 4(7.5) | 5(9.4) | 6(11.3) | 53(100) |
| Identify and practice the necessary attitudes, and skills to participate effectively in the evaluation of an outbreak. | CONFIDENT | 8(15.1) | 3(5.7) | 5(9.4) | 2(2.8) | 3(5.7) | 4(7.5) | 3(5.7) | 2(2.8) | 4(7.5) | 34(64.2) |
|  | VERY CONFIDENT | 1(1.9) | 3(5.7) | 1(1.9) | 0(0) | 5(9.4) | 0(0) | 0(0) | 3(5.7) | 2(2.8) | 15(28.3) |
|  | NOT CONFIDENT | 1(1.9) | 0(0) | 1(1.9) | 1(1.9) | 0(0) | 0(0) | 1(1.9) | 0(0) | 0(0) | 4(7.5) |
| Total |  | 10(18.9) | 6(11.3) | 7(13.2) | 3(5.7) | 8(15.1) | 4(7.5) | 4(7.5) | 5(9.4) | 6(11.3) | 53(100) |
| Define the roles, responsibilities, and 'ways of thinking' for the various disciplines and professions involved in improving global health. | CONFIDENT | 6(11.3) | 3(5.7) | 2(2.8) | 2(2.8) | 3(5.7) | 4(7.5) | 3(5.7) | 3(5.7) | 5(9.4) | 31(58.5) |
|  | VERY CONFIDENT | 2(2.8) | 3(5.7) | 2(2.8) | 1(1.9) | 5(9.4) | 0(0) | 1(1.9) | 2(2.8) | 1(1.9) | 17(32.1) |
|  | NOT CONFIDENT | 2(2.8) | 0(0) | 3(5.7) | 0(0) | 0(0) | 0(0) | 0(0) | 0(0) | 0(0) | 5(9.4) |
| Total |  | 10(18.9) | 6(11.3) | 7(13.2) | 3(5.7) | 8(15.1) | 4(7.5) | 4(7.5) | 5(9.4) | 6(11.3) | 53(100) |
| Work in teams to dev & practice leadership & management skills involved in addressing emerging p h challenges. | Confident | 6(11.3) | 3(5.7) | 4(7.5) | 1(1.9) | 3(5.7) | 4(7.5) | 4(7.5) | 3(5.7) | 5(9.4) | 33(62.3) |
|  | Very confident | 2(2.8) | 3(5.7) | 3(5.7) | 2(2.8) | 5(9.4) | 0(0) | 0(0) | 2(2.8) | 1(1.9) | 18(34) |
|  | Not confident | 2(2.8) | 0(0) | 0(0) | 0(0) | 0(0) | 0(0) | 0(0) | 0(0) | 0(0) | 2(2.8) |
| Total |  | 10(18.9) | 6(11.3) | 7(13.2) | 3(5.7) | 8(15.1) | 4(7.5) | 4(7.5) | 5(9.4) | 6(11.3) | 53(100) |
| Explore the evolution of an emerging epidemic from a variety of learning environments, including animal, environmental, human, and public health perspectives. | Confident | 7(13.2) | 3(5.7) | 5(9.4) | 2(2.8) | 3(5.7) | 4(7.5) | 3(5.7) | 2(2.8) | 5(9.4) | 34(64.2) |
|  | Very confident | 1(1.9) | 3(5.7) | 2(2.8) | 1(1.9) | 5(9.4) | 0(0) | 1(1.9) | 3(5.7) | 1(1.9) | 17(32.1) |
|  | Not confident | 2(2.8) | 0(0) | 0(0) | 0(0) | 0(0) | 0(0) | 0(0) | 0(0) | 0(0) | 2(2.8) |
| Total |  | 10(18.9) | 6(11.3) | 7(13.2) | 3(5.7) | 8(15.1) | 4(7.5) | 4(7.5) | 5(9.4) | 6(11.3) | 53(100) |

Supplementary Table iV. Particiopants post training assessment.

|  |  | H H | Ornit | vet med | Envir Scie | Ento | Soc Sci | Micro | PH | other |  |
| --- | --- | --- | --- | --- | --- | --- | --- | --- | --- | --- | --- |
| Social, economic, environmental, and cultural determinants of population health have on the emergence of novel pathogens. | confident | 7(13.5) | 3(5.8) | 3(5.8) | 1(1.9) | 2(3.8) | 1(1.9) | 2(3.8) | 3(5.8) | 2(3.8) | 24(46.2) |
|  | Very confident | 3(5.8) | 3(5.8) | 4(7.7) | 2(3.8) | 5(9.6) | 3(5.8) | 2(3.8) | 2(3.8) | 2(3.8) | 26(50) |
|  | Not confident | 0(0) | 0(0) | 0(0) | 0(0) | 1(1.9) | 0(0) | 0(0) | 0(0) | 1(1.9) | 2(3.8) |
| Total |  | 10(19.2) | 6(11.5) | 7(13.5) | 3(5.8) | 8(15.4) | 4(7.7) | 4(7.7) | 5(9.6) | 5(9.6) | 52(100) |
| Identify and practice the necessary attitudes, and skills to participate effectively in the evaluation of an outbreak. | CONFIDENT | 8(15.4) | 3(5.8) | 5(9.6) | 1(1.9) | 4(7.7) | 2(3.8) | 1(1.9) | 3(5.8) | 3(5.8) | 30(57.7) |
|  | VERY CONFIDENT | 1(1.9) | 3(5.8) | 2(3.8) | 2(3.8) | 4(7.7) | 2(3.8) | 3(5.8) | 2(3.8) | 2(3.8) | 21(40.4) |
|  | NOT CONFIDENT | 1(1.9) | 0(0) | 0(0) | 0(0) | 0(0) | 0(0) | 0(0) | 0(0) | 0(0) | 1(1.9) |
| Total |  | 10(19.2) | 6(11.5) | 7(13.5) | 3(5.8) | 8(15.4) | 4(7.7) | 4(7.7) | 5(9.6) | 5(9.6) | 52(100) |
| Define the roles, responsibilities, and 'ways of thinking' for the various disciplines and professions involved in improving global health. | CONFIDENT | 7(13.5) | 2(3.8) | 5(9.6) | 1(1.9) | 4(7.7) | 3(5.8) | 2(3.8) | 3(5.8) | 3(5.8) | 30(57.7) |
|  | VERY CONFIDENT | 2(3.8) | 4(7.7) | 2(3.8) | 2(3.8) | 4(7.7) | 1(1.9) | 2(3.8) | 2(3.8) | 2(3.8) | 21(40.4) |
|  | NOT CONFIDENT | 1(1.9) | 0(0) | 0(0) | 0(0) | 0(0) | 0(0) | 0(0) | 0(0) | 0(0) | 1(1.9) |
| Total |  | 10(19.2) | 6(11.5) | 7(13.5) | 3(5.8) | 8(15.4) | 4(7.7) | 4(7.7) | 5(9.6) | 5(9.6) | 52(100) |
| Work in teams to develop and practice the fundamental leadership and management skills involved in addressing emerging public health challenges. | confident | 4(7.7) | 2(3.8) | 1(1.9) | 1(1.9) | 4(7.7) | 1(1.9) | 1(1.9) | 2(3.8) | 3(5.8) | 19(36.5) |
|  | very confident | 5(9.6) | 4(7.7) | 6(11.5) | 2(3.8) | 4(7.7) | 3(5.8) | 3(5.8) | 3(5.8) | 2(3.8) | 32(61.5) |
|  | Not confident | 1(1.9) | 0(0) | 0(0) | 0(0) | 0(0) | 0(0) | 0(0) | 0(0) | 0(0) | 1(1.9) |
|  |  | 10(19.2) | 6(11.5) | 7(13.5) | 3(5.8) | 8(15.4) | 4(7.7) | 4(7.7) | 5(9.6) | 5(9.6) | 52(100) |
| Explore the evolution of an emerging epidemic from a variety of learning environments, including animal, environmental, human, and public health perspectives. | confident | 3(5.8) | 3(5.8) | 3(5.8) | 1(1.9) | 3(5.8) | 3(5.8) | 3(5.8) | 2(3.8) | 3(5.8) | 24(46.2) |
|  | very confident | 6(11.5) | 3(5.8) | 4(7.7) | 2(3.8) | 5(9.6) | 1(1.9) | 1(1.9) | 3(5.8) | 2(3.8) | 27(51.9) |
|  | Not confident | 1(1.9) | 0(0) | 0(0) | 0(0) | 0(0) | 0(0) | 0(0) | 0(0) | 0(0) | 1(1.9) |
| Total |  | 10(19.2) | 6(11.5) | 7(13.5) | 3(5.8) | 8(15.4) | 4(7.7) | 4(7.7) | 5(9.6) | 5(9.6) | 52(100) |
